# Interventions to Modify Psychological Well-Being: Progress, Promises, and an Agenda for Future Research

**DOI:** 10.1007/s42761-022-00167-w

**Published:** 2023-03-03

**Authors:** Laura D. Kubzansky, Eric S. Kim, Julia K. Boehm, Richard J. Davidson, Jeffrey C. Huffman, Eric B. Loucks, Sonja Lyubomirsky, Rosalind W. Picard, Stephen M. Schueller, Claudia Trudel-Fitzgerald, Tyler J. VanderWeele, Katey Warran, David S. Yeager, Charlotte S. Yeh, Judith T. Moskowitz

**Affiliations:** 1 Department of Social & Behavioral Sciences, Lee Kum Sheung Center for Health and Happiness, Harvard T. H. Chan School of Public Health, Boston, MA USA; 2 Department of Psychology, University of British Columbia, Vancouver, Canada; 3 Department of Psychology, Chapman University, One University Drive, Orange, CA USA; 4 Center for Healthy Minds, University of Wisconsin-Madison, Madison, WI USA; 5 Department of Psychiatry, Massachusetts General Hospital, Boston, MA USA; 6 Department of Psychiatry, Harvard Medical School, Boston, MA USA; 7 Department of Epidemiology, Mindfulness Center, Brown University School of Public Health, Providence, RI USA; 8 Department of Psychology, University of California, Riverside, Riverside, CA USA; 9 MIT Media Lab, MIT Cambridge MA and Empatica, Inc., Boston, MA USA; 10 Department of Psychological Science, University of California, Irvine, Irvine, CA USA; 11 Department of Psychology, Université du Québec À Trois-Rivières, Trois-Rivières, Canada; 12 Research Center, Institut Universitaire en Santé Mentale de Montréal, Montreal, Canada; 13 Departments of Epidemiology and Biostatistics, Harvard T. H. Chan School of Public Health, Boston, MA USA; 14 Human Flourishing Program, Harvard University, Cambridge, MA USA; 15 Research Department of Behavioural Science and Health, WHO Collaborating Centre for Arts & Health, University College London, London, UK; 16 Department of Psychology, University of Texas at Austin, Austin, TX USA; 17 AARP Services, Inc., Washington, DC USA; 18 Department of Medical Social Sciences, Northwestern University, Chicago, IL USA

**Keywords:** Psychological well-being, Positive psychology, Health

## Abstract

Psychological well-being, characterized by feelings, cognitions, and strategies that are associated with positive functioning (including hedonic and eudaimonic well-being), has been linked with better physical health and greater longevity. Importantly, psychological well-being can be strengthened with interventions, providing a strategy for improving population health. But are the effects of well-being interventions meaningful, durable, and scalable enough to improve health at a population-level? To assess this possibility, a cross-disciplinary group of scholars convened to review current knowledge and develop a research agenda. Here we summarize and build on the key insights from this convening, which were: (1) existing interventions should continue to be adapted to achieve a large-enough effect to result in downstream improvements in psychological functioning and health, (2) research should determine the durability of interventions needed to drive population-level and lasting changes, (3) a shift from individual-level care and treatment to a public-health model of population-level prevention is needed and will require new infrastructure that can deliver interventions at scale, (4) interventions should be accessible and effective in racially, ethnically, and geographically diverse samples. A discussion examining the key future research questions follows.

Numerous studies have documented a rise in deaths of despair, deaths arising from suicide, drug overdose, and alcoholism (Case & Deaton, 2015). Despite the recognition of the critical interplay between mental and physical health, much research has focused on risk factors and deficits. Recent work, however, suggests the enormous value of examining positive health assets as well (Kubzansky et al., 2018; VanderWeele et al., 2020). Psychological well-being, characterized by feelings, cognitions, and strategies that are associated with positive functioning (including hedonic and eudaimonic well-being), is important in its own right, but accumulating evidence suggests it also uniquely contributes to better physical health and longer lives (Kubzansky et al., 2018; Levine et al., 2021; Ryff, 2014; Seligman, 2008; Steptoe, 2019; VanderWeele, 2017).^1^ For example, a 2017 meta-analysis of 76 prospective studies found higher levels of optimism, sense of purpose in life, positive affect, and life satisfaction are consistently associated with reduced mortality risk (Martín-María et al., 2017). An emerging literature has identified potential underlying mechanisms including reductions in harmful health behaviors (e.g., cigarette smoking, physical inactivity, poor diet) and biological processes (e.g., elevated inflammation) through which psychological well-being leads to health benefits (J. K. Boehm et al., 2018; Feig et al., 2022; Kim et al., 2019; Kubzansky et al., 2018; Ryff, 2014; Seligman, 2008; VanderWeele, 2017).

Because various dimensions of psychological well-being can be modified (Carr et al., 2021), investigators have proposed interventions targeting psychological well-being as one strategy for improving physical health in both the general population and individuals with medical conditions (van Agteren et al., 2021). However, such proposals rely on the assumption that these factors are not only modifiable, but also meaningful, durable, and scalable. To explore the exciting possibility that psychological well-being interventions can also contribute to improving population health, the Lee Kum Sheung Center for Health and Happiness at the Harvard T.H. Chan School of Public Health hosted a 2-day cross-disciplinary workshop, “Interventions to Modify Psychological Well-Being: What Works, What Doesn’t Work, and an Agenda for Future Research.”

This workshop builds on prior work in the Science of Behavior Change (SOBC). Starting in 2009, researchers in the SOBC network developed an understanding of how to create and sustain effective change in adaptive health behaviors such as diet, exercise, and medication adherence (Nielsen et al., 2018). The SOBC approach follows four steps: (1) identify the hypothesized mechanism underlying behavior change, (2) measure it accurately and precisely, (3) influence or intervene to impact the mechanism, and (4) evaluate if the intervention-induced changes in the mechanism translates into behavior change. Our workshop focused on psychological well-being as one mechanism that may be harnessed to drive behavior change, as well as to trigger other biobehavioral changes that lead to improved physical health. In addition, we noted that the SOBC principles outlined for changing behaviors can be applied to changing psychological well-being itself. Our overarching aim was to create a research agenda for developing and evaluating scalable psychological well-being interventions that are sufficiently powerful to improve physical health at the population level.

The workshop began with a recognition that subjective interpretations of well-being have generally been considered in the context of hedonic (i.e., involving pleasure and happiness) or eudaimonic (i.e., involving optimal psychological functioning and self-realization) well-being (Keyes et al., 2002). Workshop attendees noted that psychological well-being is separate from states of psychological distress and related disorders (e.g., absence of anxiety does not necessarily equate to high levels of joy and meaning). Thus, interventions designed to enhance psychological well-being need to go beyond simply reducing symptoms of psychological distress, and evaluation of these interventions should reflect this understanding; additionally, researchers should separate boosts in psychological well-being from decreases in distress by including and measuring both outcomes carefully. Attendees presented examples of interventions with documented success in modifying psychological well-being and then considered a set of related issues, including the magnitude of effect sizes from well-being interventions and whether these reflect changes in psychological well-being that are large enough to influence downstream health behaviors and processes, the scaling of interventions, barriers to developing durable and scalable interventions, and strategies that might overcome these barriers. Below, we summarize insights from the convening.

## Summary of the Workshop

The workshop included presentations around select interventions with demonstrated effects on psychological well-being. For example, Eric Loucks presented his work on mindfulness-based interventions, oriented toward the question of what allows people to make shifts in life to promote psychological and physical well-being (Loucks et al., 2019, 2022). Specifically, he described mindfulness-based stress reduction (MBSR) techniques tested in clinical trials, which showed that MBSR leads to improved psychological well-being (de Vibe et al., 2017) and physical health as demonstrated in more recent studies among medical populations (Loucks et al., 2019). Richard Davidson described findings demonstrating that mindfulness-based interventions can affect neuroplasticity and epigenetics and suggested these biological alterations can elucidate whether and how MBSR leads to improved physical health (Chaix et al., 2020; Davidson & McEwen, 2012). Further, based on epigenetic findings, he raised the intriguing possibility of intergenerational transmission of well-being, which could be viewed as a particularly durable intervention effect.

Jeff Huffman, Judy Moskowitz, and Sonja Lyubomirsky each discussed their research on positive psychological interventions (PPIs)—interventions that explicitly target psychological well-being (Fritz & Lyubomirsky, 2018; Lyubomirsky & Layous, 2013). Meta-analyses of PPIs demonstrate these interventions have consistent, albeit relatively modest, effects on psychological well-being (Carr et al., 2021; Koydemir et al., 2021; van Agteren et al., 2021). However, evidence that PPIs may ultimately influence physical health outcomes is more limited, likely due to time and budget constraints that restrict studies to relatively brief follow-up time and smaller sample sizes which can make it more difficult to detect effects on many more distal physical health outcomes.

Katey Warran discussed research on the role of arts-based interventions in improving health and well-being. Drawing on the 2019 WHO Health Evidence Network synthesis report, Warran defined arts activities as including performing arts activities, visual arts participation, literature engagement, digital arts activities, and cultural engagement (e.g., going to museums and galleries). She characterized the evidence regarding the benefits of the arts as substantial and described numerous observational studies that have demonstrated an arts-health association. To illustrate, she described a study that showed engaging in arts activities was associated with reduced depression (Bone et al., 2022). She further noted that arts activities are considered complex interventions because they combine multiple components to initiate non-linear mechanisms of action that influence mental and physical health outcomes (Fancourt et al., 2021; Warran et al., 2022).

Tyler VanderWeele provided an overview of forgiveness interventions (Wade & Tittler, 2019). VanderWeele modified existing interventions for large-scale application (i.e., distilling established protocols into a 2–3-h workbook that can be administered on-line or in-person) and to global audiences (e.g., Indonesia or South Africa). Current research suggests these interventions lead to increased forgiveness and hope, as well as reduced depression and anxiety (VanderWeele, 2018; Wade & Tittler, 2019). VanderWeele further noted that forgiveness interventions may also lead to higher community levels of forgiveness, which could contribute to healing community and political divides. However, research has not yet established whether these interventions are sufficiently potent to induce subsequent changes in physical health. Given the potential ease of disseminating forgiveness workbooks, such interventions may have an important role in promoting population health (VanderWeele, 2018).

Noting that numerous effective interventions already exist, Stephen Schueller expressed concern that translation into widespread dissemination has been slow. He suggested progress will require moving beyond repeated pilot and efficacy trials toward greater investment in implementation. Such efforts should include identifying facilitators and barriers to successful implementation across contexts and then using this information to inform the design and evaluation of effective implementation strategies (Bauer & Kirchner, 2020). The research agenda for PPIs should consider stage of evidence to determine unanswered questions in implementation (Lane-Fall et al., 2019) and use appropriate study designs to explore these questions (Wolfenden et al., 2021). Schueller further noted that mode of delivery is important when implementing scaled-up versions of interventions. Promising approaches include digital delivery, single-session interventions, and micro-interventions (i.e., highly focused low-burden brief interventions delivered in the context of a person’s daily life; Baumel et al., 2020; Hirshberg et al., 2022).

Based on the workshop discussions, we see several exciting directions for research to provide greater insight into whether and how we might create scalable interventions to modify psychological well-being in ways sufficiently powerful to influence downstream health outcomes. In the following section, we highlight four key topics, then discuss additional substantive issues that emerged, and conclude with thoughts on the future.

## 1) What Are Meaningful Effect Sizes?

Many existing interventions rely on either “light-touch” brief activities (e.g., writing a gratitude journal) or more intensive delivery methods (e.g., positive psychotherapy, meditation training; J. Boehm et al., 2012). Whereas “light-touch” interventions are more scalable, it remains unclear if they have sufficiently durable or potent effects to catalyze meaningful improvements in downstream health endpoints. A recent systematic review and meta-analysis of interventions designed to improve psychological well-being considered not only PPIs developed within the field of positive psychology but also non-PPIs such as mindfulness meditation and more traditional therapeutic approaches (van Agteren et al., 2021). The review found psychological well-being can be enhanced across varied interventions and effects differed according to target population (e.g., general population versus physically ill patients) and, most notably, intervention intensity (e.g., multi-component versus single element interventions). Less well studied is how large an increase in psychological well-being is needed to observe meaningful downstream effects on physical health, and this likely depends on which physical health outcomes are considered. Moreover, attendees suggested large samples may be needed to detect effects in randomized trials, particularly if intervention effects on psychological well-being and subsequent effects on physical health are both modest.

Important to note is that whereas meta-analyses show promising average effect sizes, effects are also highly heterogeneous. Understanding the heterogeneity of effects (e.g., where and with whom each intervention works) is critical for assessing whether any given effect appears small because interventions were incorrectly targeted to some subgroups or did not fully account for the context in which they occur. Without understanding this heterogeneity, it is difficult to appropriately power studies.

Ultimately, workshop attendees noted that even if changes in psychological well-being lead only to small changes in downstream behaviors and physical health, such effects are still valuable—especially if the interventions are easy to deploy, scale, and adopt at the population level. Given most health outcomes are multiply determined, any one variable likely contributes only modestly. For example, associations between aspirin and prevention of heart attacks (*r* = 0.03) or cardiac patient education and exercise (*r* = 0.09) appear small (Götz et al., 2022). However, these interventions are important because small effect sizes translate into meaningful changes at the population level under particular circumstances (e.g., if many small effects act in concert to create larger substantive composite effects) or as they accumulate across the lifespan (Götz et al., 2022).

The impact of PPIs also differs depending on context. Investigators must carefully consider the population under study and the social environment in which interventions are implemented (Bryan et al., 2021). A new “moderation as mediation” framework illustrates how contextual factors can act like a switch that turns mechanistic pathways on or off. For example, one study examined effects of a growth mindset intervention (i.e., bolstering beliefs that abilities are learnable and can be improved through effort) delivered to students on their math performance. Investigators first evaluated teachers’ mindsets. If a teacher believed that abilities are inherently stable and unchangeable, then the effect of growth mindset interventions on students’ math GPAs was minimal. However, when teachers themselves had a growth mindset, then the intervention successfully enhanced students’ performance (Yeager et al., 2022).

## 2) How Durable Are Effects of Psychological Well-Being Interventions?

If psychological well-being influences physical health, it is likely because relevant psychological states are enduring and thereby lead to recurring effects on health-relevant habits and biological processes. Interventions seeking to change psychological well-being sufficiently to impact downstream biobehavioral processes related to physical health will need to produce sustained effects. However, durability of effects is less well-understood (Miller et al., 2017). In one of the largest meta-analyses of PPIs, most studies tracked outcomes for < 6 months and none for > 12 months (Carr et al., 2021). Thus, it remains unclear if effects of PPIs are sustained over periods long enough to lead to changes in physical health. Moreover, studies including longer follow-up time will need to consider carefully how to capture the durability of intervention effects, including deciding how and at what intervals investigators should measure changes in psychological well-being, as well as how to retain study participants over longer periods.

Several substantive issues are also relevant. First, investigators should evaluate whether interventions create a habit (e.g., teach skills that become habitual to repeatedly boost effects over time) or crystallize a new way of thinking (e.g., “wise interventions” that target psychological processes contributing to core underlying thought processes and recursive dynamics that compound over time; Cohen et al., 2017; Miller et al., 2017; Walton & Wilson, 2018).

Second, developmental theory suggests there are points in the life course when exposures to certain risk factors are particularly harmful (i.e., sensitive periods like before a major life transition), and also when health interventions may be most effective (Bailey et al., 2020; Berkman, 2009; Meyer et al., 2012). Scholars should seek to identify these optimal points or “signature moments.” Of note, optimal timing for delivering interventions may also depend on which facet of psychological well-being is targeted. For example, purpose in life interventions might be particularly helpful during identity development, “midlife crises,” and retirement—destabilizing periods due to the many substantial changes in life patterns; at such times, developing or re-discovering a sense of purpose may mitigate potential derailment.

Third, if psychological well-being interventions need repeated administration for durable effects, it will be important to embed these interventions into systems and social practices. For example, clear evidence that psychological well-being interventions enhance health and reduce healthcare costs motivate healthcare systems to adopt and maintain these interventions. To facilitate development of sustainable financing models we might encourage creating healthcare system classification codes that facilitate tracking and reimbursement of evidence-based interventions.

## 3) Delivery and Scalability of Psychological Well-Being Interventions

Psychological well-being interventions that demonstrate the strongest effects are often complex (involving multiple components), time-intensive to deliver, and require in-person attendance. Moreover, following a biomedical model, many psychological well-being interventions target individuals who are either high-risk or already have disease. A key activity for future work following a public health model of prevention is to determine if existing labor-intensive interventions developed in medically high-risk populations can be adapted for use in the general population and delivered at a manageable cost. Investigators will also want to consider whether existing “light-touch” interventions, which are easier to deliver on a larger scale but often demonstrate smaller (and perhaps less durable) effects on psychological well-being, can be modified to enhance both size and durability of effects. Some work suggests rigorously optimized simpler interventions can have more durable effects, including findings from studies of single session interventions (Schleider & Weisz, 2016). Attendees also noted that “light-touch” interventions targeting domains like a sense of belonging and academic performance have shown large and durable effects (e.g., lasting 6–11 years; Brady et al., 2020; Goyer et al., 2017). Important next steps include linking these interventions to physical health.

One possible concern with shifting intervention work to the general population is that healthy individuals may be less likely to participate as they are not motivated by a specific illness or problem that needs attention. By anticipating this potential concern ahead of time, those who deliver the intervention should carefully consider how to present these interventions to the public. Further, when conducting interventions in healthy populations, the metric of health would not be survival or improvement in disease status, but rather delayed onset of disease, a more difficult outcome to capture, especially in the short term.

These concerns notwithstanding, workshop attendees noted the COVID-19 pandemic spurred development of innovative methods of synchronous and asynchronous intervention delivery that can now be leveraged to advance the field. Discussions around scalability often focus on the role of technology (e.g., smart watches, app-based delivery), and the COVID-19 pandemic drove wider adoption of these methods. Examples from behavioral health can inform thinking on how to scale PPIs. For example, a successful treatment for individuals with substance use disorders relied on administering cognitive behavioral therapy. While effective, delivering the intervention widely was not feasible due to high cost. A computerized version of the intervention was developed, and subsequent evaluations found it was equally as effective as in-person delivery (Carroll et al., 2009, 2014). Other exciting possibilities for digital interventions were also identified. For example, workshop attendees noted micro-interventions that sprinkle 30–90 s doses of intervention content throughout the day via mobile devices should be further evaluated.

Our discussion also highlighted potential pitfalls of these intervention dissemination methods. Barriers to quitting digitally delivered interventions are lower, resulting in higher drop-out. A recent review of mental health app usage by people in the real world observed that across 93 mental health apps, median retention rate at 15 days was 3.9% (Baumel et al., 2019). Newer methods of enhancing retention have somewhat improved these numbers. For example, one study of a game implementing mental health therapy in real-world conditions (7,782 users) reported 10% retention at 15 days which was a doubling of the previously reported retention rates. Despite this improvement, it is still the case that 90% of those who started therapy were not retained (Ferguson et al., 2021). Moreover, participants who drop out are often those who are most in need of the intervention or who are the least motivated, so failure to retain them can produce a biased understanding of efficacy. Digital formats may also render it more difficult to deploy features demonstrated to promote continued engagement in interventions, such as developing relationships with participants. Finally, some interventions may not translate easily to digital formats resulting in a loss of their potency.

Workshop attendees reiterated the value of creating a “science of engagement” to help identify not only different profiles of intervention participants (digital or otherwise) but also an accounting of active ingredients and optimal dosages that work best for different individuals. Investigators should consider when and how interventions can incorporate elements of fun to promote retention and continued engagement. For all modes of intervention, interpersonal connection or “human touch” is likely critical for promoting and maintaining engagement. Investigators should consider how they might embed interventions into the existing infrastructure of relationships individuals already have. For example, studies could capitalize on existing social relationships to enhance social support, collaboration, or competition (e.g., Patel et al., 2021). Additionally, in the realm of healthcare, researchers might work with general practitioners, nurses, or social workers who could then provide their clients with strategies to improve psychological well-being (see Kubzansky et al., 2018). This type of engagement could lower participation barriers for people who are less digitally connected.

## 4) Do Psychological Well-Being Interventions Work in Diverse Populations and Settings?

Tailoring and contextualizing interventions for minoritized and underserved populations are critical. The same intervention may work differently depending on gender, race, ethnicity, and other characteristics of the population. Interventions are less often tested in disadvantaged individuals including those with severe financial constraints, limited access to technology, or extreme time scarcity. Interventions developed for individuals who are already ill may need substantial adaptation (or simply not apply) for use with healthy individuals for whom the ultimate goal is to *prevent* illness. Interventions can backfire when administered in settings and populations for which assigned activities have poor “fit.” For example, gratitude interventions can be problematic when delivered to members of cultures where gratitude is experienced as indebtedness or guilt, or to severely depressed individuals, for whom expressions of gratitude can increase feelings of being a burden on others (Fritz & Lyubomirsky, 2018).

Prior to implementing any intervention on a large scale, investigators should gain as much insight as possible into the populations targeted for intervention. One strategy for doing this might be to conduct pre-implementation focus groups with members of the community and other key stakeholders; these can provide critical information regarding fit of a planned intervention with the target population. Administrative data (i.e., zipcode, neighborhood characteristics) and more detailed individual-level data may also be needed to design interventions that are appropriate and relevant. Efforts to obtain this kind of information are particularly important when developing interventions for traditionally minoritized or underserved groups for whom fewer interventions have been developed, and whose needs are less well understood by most investigators. To ensure interventions are both effective and sustainable, investigators will need to work directly with these groups through an iterative process to create protocols and principles that most directly serve the group’s needs (Hernandez et al., 2016, 2018; Lau, 2006).

Investigators will also need to move away from convenience samples and use varied strategies to increase the likelihood that individuals in specific populations participate. A key approach will be to develop novel ways to integrate interventions into everyday contexts and develop partnerships with institutions like schools, workplaces, or healthcare settings. Other possibilities include implementing interventions through partnerships with health insurance companies or other organizations (e.g., older adult advocacy groups like AARP). Such partnerships may increase the sustainability of interventions for continued implementation in the real world. Several workshop attendees provided examples of successful partnerships. For example, effective interventions developed and tested in the Army (e.g., Army Wellness Centers) have had broad reach across soldiers, their family members, retirees, and others. However, non-academic partners may have the sense that researchers are less interested in taking the time to gain the expertise and competence needed to work with specific groups or on questions that are of direct interest to the community. For academics, it can be difficult to ensure sufficient rigor and transparency in the research, advance scientific knowledge in non-proprietary ways, and align timelines with their non-academic partners.

## Additional Research Issues

Several cross-cutting research issues were also identified. A key debate is the conceptualization and measurement of psychological well-being, as well as the content and contours of psychological well-being relative to other factors (see EWB article this issue). It will be important to seek consistency in outcome assessment across studies (Moskowitz et al., 2021). Second, researchers might consider identifying a core set of well-being questions that can be applied consistently across studies. This would make it easier to compare results for different interventions conducted in different populations and settings. Another way to enhance comparability across studies is to harness the “megastudy” experimental paradigm, in a massive field experiment where many different treatments are tested synchronously in one large sample using a common, objectively measured outcome. For example, a consortium of 30 scientists from 15 different universities worked in small independent teams to design and test a total of 54 unique digital interventions aimed at promoting gym attendance among 61,293 members of an American fitness chain (Milkman et al., 2021). This experimental paradigm has several advantages including: (1) the ability to compare diverse interventions in an “apples-to-apples” manner by reducing the inherent heterogeneity that arises when studies are conducted independently, (2) enabling economies of scale, and (3) accelerating the pace of science (e.g., enhancing the ability to publish null results).

Third, creating optimal control groups presents significant challenges (Freedland et al., 2011). It is often difficult to create an activity for the control group that permits isolating the active ingredient of an intervention. For example, a study aiming to demonstrate that engaging in prosocial acts leads to improved psychological well-being would need to assess if prosociality per se is the active ingredient, versus simply engaging in a social activity (Regan et al., In Press). An appropriate control activity would have participants perform acts that are social but not prosocial; however, identifying clear boundaries between similar activities can be difficult.

Fourth, managing expectations in control groups is critical, as is measuring participant beliefs and expectations to understand how intervention and control procedures are received (Haeck et al., 2016). To make these more nuanced comparisons, larger samples are often needed, but this raises additional dilemmas regarding resource allocation. It may not be clear if comparing an intervention to a sham intervention or active control is better than running competing interventions against each other to establish which is more effective (Hameiri & Moore-Berg, 2022). For example, one study compared effects of a PPI (i.e., writing about three things that went well each day, for seven days) with a positive placebo (i.e., writing about a positive memory for seven days) and found no difference in depression between the intervention and placebo, although some differences in happiness were evident (Mongrain & Anselmo-Matthews, 2012). One challenge with this comparison is that writing about a positive memory may also be considered a PPI. More broadly, this work suggests the importance of carefully considering the content of control conditions that allow for differentiating between positive interventions and positive expectancies.

A fifth issue relates to whether and which specific dimensions of psychological well-being should be targeted when aiming to enhance physical health. A recent narrative review evaluated articles examining various facets of well-being in relation to mortality, including only those that featured large sample sizes with robust adjustment for covariates; purpose in life, optimism, and life satisfaction were most consistently associated with reduced mortality risk, independent of covariates, followed by ikigai, positive affect, mastery, and sense of coherence (Trudel-Fitzgerald et al., 2021). The review found inconsistent relationships of mortality with happiness, personal growth, and autonomy, or there was too little research to draw firm conclusions. Ideally, future research will be able to include these facets within the same study and then compare effect estimates of each dimension of psychological well-being on the health outcome of interest to determine if some dimensions should be prioritized for intervention. Future research might also consider whether dimensions of psychological well-being that have not yet been considered in relation to physical health (e.g., self-acceptance, joy, awe) are promising (Trudel-Fitzgerald et al., 2021). In fact, with regard to interventions seeking to modify psychological well-being, many studies have targeted overall well-being non-specifically (e.g., via cash transfers; Dwyer & Dunn, 2022; Kushlev et al., 2020; McGuire et al., 2022). Whether gains achieved by improving a specific facet of psychological well-being in relation to physical health are greater than simply aiming to improve overall well-being (or any single facet) has not yet been determined. However, some research suggests that targeted approaches may be more effective (Trudel-Fitzgerald et al., 2021).

Sixth, it is also critical to evaluate mechanisms by which interventions lead to improved psychological well-being. Studies may want to consider repeated assessments of psychological states over time as well as measures of potential mediators or explanatory factors to facilitate tests of mediation. Recent work suggests the value of considering *dynamic processes* as mechanisms or pathways. For example, a recent paper identified a set of dynamic psychosocial processes through which PPIs may enhance psychological well-being, including: (1) attention and awareness, (2) comprehension and coping, (3) emotions, (4) goals and habits, and (5) virtues and relationships (Rusk & Waters, 2015). Such work can guide for future work seeking to examine and test mechanisms of PPIs more explicitly. Similar work can be done to identify mechanisms by which emotional well-being improves physical health, another critical issue for the field.

Several additional activities will further enhance progress. Investigators should explicitly evaluate whether some features of interventions make them more robust to inevitable differences in how they are implemented in real-world settings. Administering combinations of single interventions may be more effective than administering any one singly. Exploring methodological innovations may also help address limitations in existing studies. For example, integrative data analysis is a recently developed method by which studies using similar approaches can be combined (Graham et al., 2022). Applying these methods will allow for greater power to detect small effects.

## What Does the Future Hold

After an intensive and rich exchange of ideas, workshop attendees identified several important lessons that inform next steps. They noted the need for sustained funding to support this research. While the NIH SOBC initiative has been an important source of funding, investigators also need to develop other partnerships. Foundations may have more appetite for piloting interventions that have “good enough” evidence to accelerate what is currently a long timeline for taking interventions from development to demonstrations of efficacy and implementation. Elevating the importance of the issues identified in the workshop regarding effect sizes, durability, and scalability will also be useful.

Diverse and transdisciplinary teams are needed for this work to succeed. Ideally, teams would possess diverse lived experiences and viewpoints, gather experts in basic science, intervention research, implementation science, and complexity science and incorporate input from key stakeholders to inform development of the most effective interventions. Such teams may be easier to find and build with the shareable resources and the creation of networks. Further discussion revolved around increasing political will to invest in psychological well-being. One way would be to survey public opinion and attitudes on psychological well-being and its importance in health and leverage the findings to engage and build partnerships with policy-makers and economists. Several other countries have developed high level positions aimed at fostering greater policy engagement with research in this domain, including the United Arabic Emirates and the UK, and U.S. Surgeon General Vivek Murthy is advocating this approach (Murthy, 2022).

Another strategy may be to link psychological well-being to other endpoints of significant concern to policy makers and funders. For example, recent work has identified the economic value of targeting and delaying aging (Scott et al., 2021). Additionally, the psychopathology literature has estimated the economic costs of several psychological illnesses and the potential cost savings by implementing interventions that aim to decrease these ailments (Knapp & Wong, 2020). More generally, research in this area might adopt methods developed in other domains using cost-effectiveness analysis to prioritize intervention strategies based not only on cost and effectiveness, but also on feasibility and projected population impact. For example, research on physical activity promotion and obesity prevention in childhood has taken this economic evaluation approach, first using a systematic review to identify key interventions with evidence of effectiveness and then using microsimulation models of the U.S. population to project effects of nationally implementing each intervention on physical activity and childhood obesity, drawing on current population health and health care cost data (Cradock et al., 2017). Similar methods might be effectively applied to evaluating interventions to modify psychological well-being. Knowing how psychological well-being might impact trajectories of aging and costs would generate interest in investment and sustainable funding for interventions to increase population-levels of psychological well-being. More generally, attendees agreed that given current trends in population health and particularly mental health, it is more important than ever to think creatively about ways to improve psychological well-being at the population level. We hope that sharing this discussion and the creativity, energy, and passion in the meeting will inspire continued work in this area and draw new investigators at all levels to the endeavor.

## Appendix

Workshop Participants.

Julia Boehm, Chapman University.

Ruijia Chen, University of California, San Francisco.

Richard Davidson, University of Wisconsin-Madison.

Jeffrey Huffman, Massachusetts General Hospital.

Eric Kim, University of British Columbia.

Laura Kubzansky, Harvard T.H. Chan School of Public Health.

Eric Loucks, Brown University.

Sonja Lyubomirsky, University of California, Riverside.

Wendy Mendes, University of California, San Francisco.

Judy Moskowitz, Northwestern University Fienberg School of Medicine.

Lis Nielsen, National Institute on Aging.

Lisa Onken, National Institute on Aging.

Rosalind Picard, MIT Media Lab.

Alonzo Plough, Robert Wood Johnson Foundation.

Theresa Santo, U.S. Army Public Health Center.

Stephen Schueller, University of California, Irvine.

Claudia Trudel-Fitzgerald, Université du Québec à Trois-Rivières and Research Center of Institut Universitaire en Santé Mentale de Montréal.

Tyler VanderWeele, Harvard T.H. Chan School of Public Health.

Vish Viswanath, Harvard T.H. Chan School of Public Health.

Katey Warran, University College London.

David Yeager, University of Texas, Austin.

Charlotte Yeh, AARP.

## Footnotes

1 We note ongoing discussion about terminology; the workshop was convened using the term “psychological well-being.” However, since the convening, investigators have proposed using the term “emotional well-being” when discussing a similar construct. For the current article, we continue using the term “psychological well-being” as this is the term used in the convening, but refer readers to the article Park et al., 2023, describing the use of the term “emotional well-being.”.
